# Tunable Optical Nanoantennas Incorporating Bowtie Nanoantenna Arrays with Stimuli-Responsive Polymer

**DOI:** 10.1038/srep18567

**Published:** 2015-12-18

**Authors:** Qiugu Wang, Longju Liu, Yifei Wang, Peng Liu, Huawei Jiang, Zhen Xu, Zhuo Ma, Seval Oren, Edmond K. C. Chow, Meng Lu, Liang Dong

**Affiliations:** 1 Department of Electrical and Computer Engineering, Iowa State University, Ames, Iowa 50011, USA; 2 Department of Aerospace Engineering, Iowa State University, Ames, Iowa 50011, USA; 3 Micro and Nanotechnology Laboratory, University of Illinois at Urbana-Champaign, Urbana, Illinois 61801, USA; 4 Department of Mechanical Engineering, Iowa State University, Ames, Iowa 50011, USA

## Abstract

We report on a temperature-responsive tunable plasmonic device that incorporates coupled bowtie nanoantenna arrays (BNAs) with a submicron-thick, thermosensitive hydrogel coating. The coupled plasmonic nanoparticles provide an intrinsically higher field enhancement than conventional individual nanoparticles. The favorable scaling of plasmonic dimers at the nanometer scale and ionic diffusion at the submicron scale is leveraged to achieve strong optical resonance and rapid hydrogel response, respectively. We demonstrate that the hydrogel-coated BNAs are able to sense environmental temperature variations. The phase transition of hydrogel leads to 16.2 nm of resonant wavelength shift for the hydrogel-coated BNAs, whereas only 3 nm for the uncoated counterpart. The response time of the device to temperature variations is only 250 ms, due to the small hydrogel thickness at the submicron scale. The demonstration of the ability of the device to tune its optical resonance in response to an environmental stimulus (here, temperature) suggests a possibility of making many other tunable plasmonic devices through the incorporation of coupled plasmonic nanostructures and various environmental-responsive hydrogels.

Active plasmonic devices have attracted much attention, because of an increasing demand for tunable optical properties to accommodate flexible application requirements. Often, these tunable devices are structurally variable, or hybridizing functional materials (e.g., liquid crystal, semiconductor, phase-change media, and etc) with plasmonic structures^1^,^2^. Various tuning mechanisms (e.g., mechanical stretching^3^, thermo- and electro-mechanical^4^,^5^,^6^, electro-, magneto-, and thermo-optical^7^,^8^,^9^,^10^, and electron beam manipulation^11^) were studied to regulate their structural configurations or refractive indices of surrounding media. Recently, stimuli-responsive, surface-bound hydrogels have been suggested as a promising candidate to realize active plasmonic devices^12^,^13^,^14^,^15^,^16^,^17^,^18^,^19^,^20^,^21^,^22^,^23^,^24^. These polymers are sensitive to different stimuli (e.g., temperature, pH, light, glucose, electric field, and ions strength) by changing their volume or shape^25^,^26^. Most of the existing efforts in active plasmonics with hydrogels are mainly focused on using metallic nanoparticles or islands attached to polymer brushes^12^,^13^,^14^,^15^,^16^,^17^,^18^,^19^, and on functionalizing gold (Au) films with hydrogels^20^,^21^. As these nonlithographic nanoparticles have relatively poor control over their shape and size, fine tuning for optical properties of the nanoparticles-hydrogel composites is challenging; also, their optical responses usually have undesirable broad resonance bands. Lithographically nanopatterned particles have thus been utilized to integrate with hydrogel^21^,^22^,^23^,^24^, but almost all the reported research dealt with isolated nanoparticles unfavorable to achieving high field enhancement, thus hindering the improvement in their tuning range and sensitivity to specific environmental changes.

We herein report on a temperature-responsive coupled plasmonic bowtie nanoantennas (BNAs) device capable of tuning its resonance properties in response to changing environmental conditions. In contrast to individual nanoparticles, coupled plasmonic nanoparticles provide an intrinsically higher field enhancement. Therefore, the integration of the BNAs with stimuli-responsive hydrogel is expected to bring a synergistic effect to improve tuning of active plasmonics in response to environmental changes. Basically, plasmonic BNA is coupled metallic nanoparticle dimers with two tip-to-tip nanotriangles^27^. The nanoscale air gap between the nanotriangles allows for tight confinement and large enhancement of optical fields through the excitation of surface plasmons (SPs). This effect has been harnessed for many applications, such as high-harmonic generation^28^, florescence enhancement^29^, nanolasing^30^, and optical trapping^31^,^32^. In this study the thermally tunable BNAs are formed by simply coating the top surface of BNAs with a submicron-thick, thermosensitive hydrogel. As a local environmental temperature changes, there will be a change in the refractive index of hydrogel, accompanied by swelling or deswelling behavior of the hydrogel cross-linked network in water. This will result in changing optical characteristics of the hydrogel-coated BNAs. Herein, the scaling of plasmonic dimers and ionic diffusion is favorably leveraged to achieve strong BNA resonance and rapid hydrogel response time, respectively. The creation of the temperature-responsive BNAs takes advantages of these scaling properties.

## Results and Discussion

We demonstrate the stimuli-responsive BNAs using thermosensitive poly(*N*-isopropylacrylamide) or PNIPAAm hydrogel that expands at low temperatures and contracts at high temperatures with a volume phase transition temperature (VPTT) at approximate 32 °C. The volumetric change of hydrogel causes a continuous and reversible change in its refractive index, typically between 1.36 and 1.46^33^. As the degree of swelling drastically changes around the VPTT, the hydrogel-coated BNAs present a considerable resonant wavelength shift of 16.2 nm. In contrast, with the same temperature change, the uncoated device yields only 3 nm resonant wavelength shift. Furthermore, the hydrogel-coated BNAs respond to environmental changes rapidly within 250 ms because the thickness of hydrogel is reduced to a submicron scale for fast ion diffusion. The present BNAs device is structurally simple and can be modified to incorporate many other hydrogels that respond, for example, to light, pH, electric fields, and antigens, for use as physical, biological or chemical sensors.

**Figure 1:**
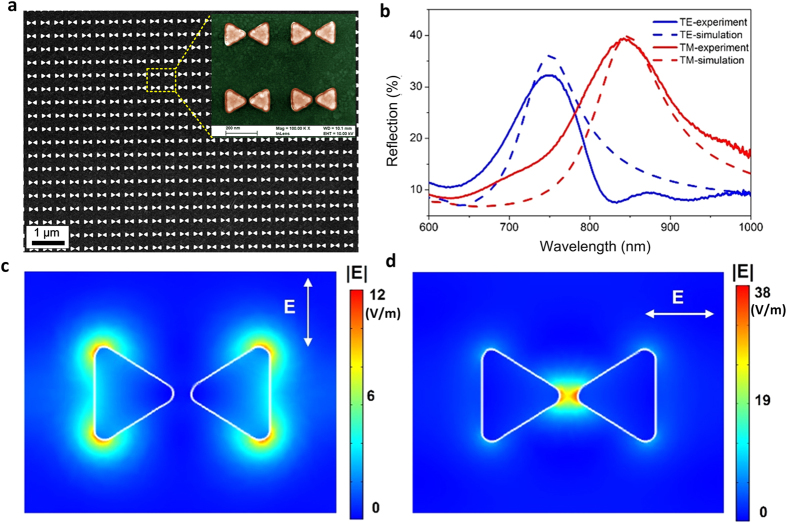
(**a**) Scanning electron microscopy (SEM) image for the bare Au BNAs without a hydrogel coating. The inset shows a pseudo-color SEM image for a close-up of BNAs. (**b**) Experimental and simulated reflection spectra of the uncoated BNAs in water under normally incident TE and TM polarized light. (**c,d**) Normalized electric field distributions at the resonances under the TE (**c**) and TM (**d**) polarization, respectively. The color scale bars show the normalized electric field amplitude relative to the incident field |**E**_*0*_|.

In this study, 50 nm thick Au BNAs were patterned in 428 nm spaced square arrays covering an area of 500 × 500 μm^2^ on a 25 nm thick indium tin oxide (ITO) coated glass substrate (Fig. 1a). Each bowtie consists of two equilateral triangles with a side length of 150 nm and a tip-to-tip distance of 20 nm (see the inset of Fig. 1a). We first measured the reflection spectra of the bare BNAs (without hydrogel coating) using a spectroscopic measurement setup. For the transverse magnetic (TM) polarization, the excitation light has the electric field component along (parallel to) the nanogap direction of the bowtie. Figure 1b shows that the uncoated BNAs have a resonance at 838 nm for TM polarization and the other at 750 nm for transverse electric (TE) polarization. We then performed full wave simulation using a finite element analysis method. The simulation results show a good agreement with the experimental ones in terms of their resonance positions (Fig. 1b). The minor difference in the spectra may be attributed to imperfect structural uniformity of the fabricated device. Figure 1c,d show the electric field distributions at the resonances under TE and TM polarizations. In the case of TM polarization, the surface plasmon resonance leads to significant field confinement inside the nanogap of the bowtie with a maximum amplitude enhancement factor of 38, while under TE polarization, the “hot spots” occur at the two base corners of each triangle with a much lower maximum enhancement factor of 12. Therefore, the strong ability of the BNAs to enhance the local field amplitude, especially at the TM resonance, is promising to enable effective tuning of their optical characteristics by minute changes of the surrounding index.

**Figure 2:**
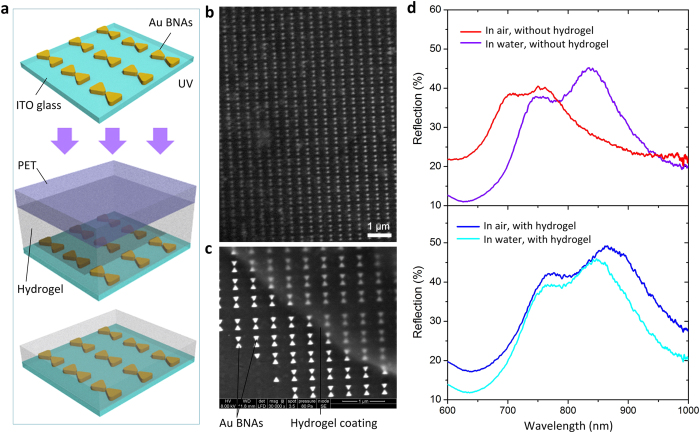
(**a**) Fabrication processes for the temperature-responsive BNAs. (**b**) SEM image of the hydrogel-coated BNAs. (**c**) SEM image showing the morphological difference between the hydrogel-coated and the uncoated BNAs. The hydrogel at the edge was intentionally unexposed to ultraviolet (UV) light during the device fabrication. (**d**) Reflection spectra of the uncoated BNAs (upper panel) and the hydrogel-coated device (lower panel) in air and water at 22 °C under non-polarized normal light incidence.

To form the proposed BNAs, we coated the top surface of the BNAs with a 750 nm thick PNIPAAm hydrogel layer^34^ (Fig. 2a–c). The fabrication details are described in Materials and Methods. We studied optical responses of the hydrogel-coated and uncoated BNAs to local environmental temperature changes. Figure 2d shows the reflection spectra of the two devices at room temperature (22 °C), both with normal incidence of non-polarized light. Immersing the uncoated BNAs in water caused a resonant wavelength red shift of 83 nm and 40 nm to the TM and TE modes, respectively. After the uncoated device were coated with PNIPAAm hydrogel, the TM and TE resonances red shifted by 92 nm and 50 nm, respectively. By immersing the hydrogel-coated device in water at 22 °C, both the TE and TM resonances red shifted, but with different amounts: 25 nm for the TM mode and 7.5 nm for the TE mode. It is also noteworthy that when immersed in water, the two devices shifted their resonant wavelength in an opposite direction. This is because the as-polymerized hydrogel on the BNAs initially absorbed water to reach an initial equilibrium, giving rise to an increase in physical volume, in accompany with a decrease in refractive index, thus causing a blue resonance shift. In addition, the introduction of the hydrogel to the surface of the BNAs did not significantly influence the bandwidth of the plasmonic resonances.

**Figure 3:**
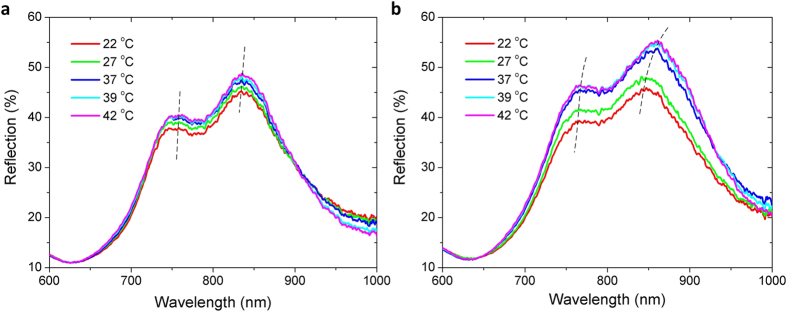
(**a**) Reflection spectra of the uncoated BNAs at different temperatures. (**b**) Reflection spectra of the hydrogel-coated BNAs at different temperatures. The spectra were measured under normally incident non-polarized light.

**Figure 4:**
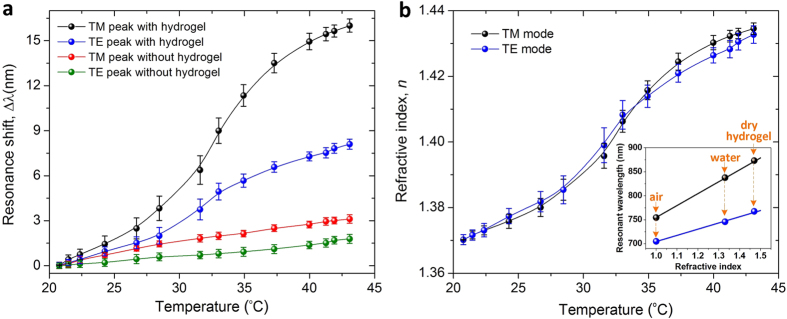
(**a**) TM and TE mode resonance shifts of the hydrogel-coated and uncoated BNAs as a function of temperature. (**b**) Calculated refractive index of the PNIPAAm hydrogel as a function of temperature. The inset shows the TE and TM resonant wavelengths of the BNAs as a function of environmental refractive index *n*. The yellow arrows indicate the different surrounding media, including air, water, and dry hydrogel^35^,^36^.

Figure 3 shows the reflection spectra of the hydrogel-coated and uncoated BNAs as the environment temperature changes from 22 to 42 °C. First, when responding to an increase in temperature, the hydrogel-coated device showed a larger increase in reflection intensity than the uncoated counterpart, because of a larger increase in refractive index for the hydrogel-coated BNAs. As for the resonance response to increasing temperature, the TM resonance peak of the hydrogel-coated device significantly red shifted by 16.2 nm, while the TE resonance peak shifted by 8 nm (Fig. 3b). This difference may result from the higher field enhancement factor at the TM mode than that at the TE mode. Figure 4a shows that the majority of the resonance shift occurred around the VPTT of the hydrogel, due to the phase transition induced large index change, confirming the function of the hydrogel in tuning the optical properties of the hydrogel-coated BNAs.

To estimate how the refractive index of the hydrogel coating changed with temperature, we first plotted the TM and TE resonant wavelengths of the Au BNAs with respect to the refractive indices of different surrounding media, including air (*n* = 1), water (*n* = 1.33), and a dry hydrogel layer (*n* = 1.48)^35^,^36^. The slopes of the two plots in the inset of Fig. 4b indicate that the BNAs have the refractive index sensitivity of 248 nm/RIU (RIU: refractive index unit) and 129 nm/RIU for the TM and TE resonances, respectively. Based on the resonance wavelength shift (Fig. 4a) of the hydrogel-coated BNAs and the refractive index sensitivity obtained above, the refractive index of the hydrogel coating at different temperatures was extracted. As the temperature increased from 22 °C to 42 °C, the refractive index increased from 1.37 to 1.435, with a total index variation of 0.065 (Fig. 4b). Essentially, the effective refractive index variation of the BNAs may be attributed to the following factors. The dominating factor is the volume change induced index change of the hydrogel coating. As the local temperature increased by 20 °C, the refractive index of the hydrogel was significantly increased by 4.74%. Another factor relates to changes in thermophysical properties of other device materials (i.e., water, Au, and ITO-coated glass), which is considered to have an insignificant effect on the resonance shift, as evident by a maximum 3 nm and 1.6 nm shift of the TM and TE mode resonance peaks, respectively, of the uncoated BNAs (Figs 3a and 4a). The temperature induced dispersion change of Au may also contribute to the observed resonance shift of both the hydrogel-coated device and the uncoated counterpart, in accompany with a minor decrease in quality factor. At 830 nm near the TM mode resonance of the BNAs, as the temperature increases from 22 °C to 42 °C, the real part of Au permittivity remains almost the same at the value of −8, while the imaginary part changes from 1.6 to 1.9^37^, which leads to an increase in radiative losses^37^. This, in turn, may cause a decrease in collective coupling of neighboring bowties, thus slightly red shifting the TM mode resonant wavelength.

**Figure 5:**
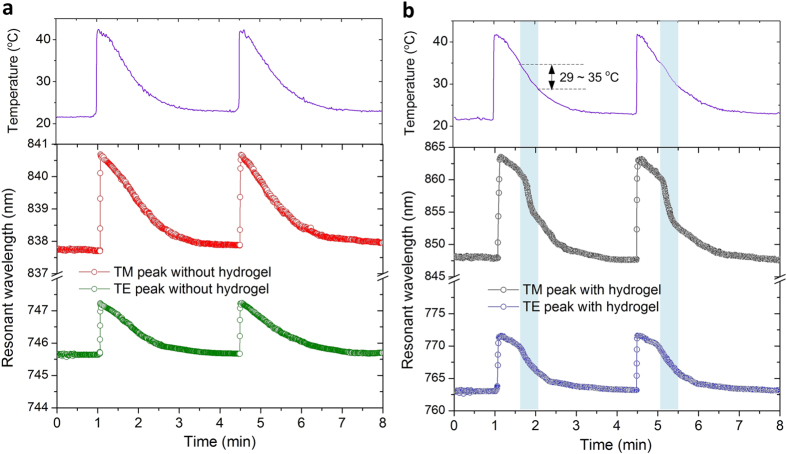
Dynamic tracking of TE and TM mode resonance peaks for the uncoated BNAs (a) and the hydrogel- coated BNAs (b) at different temperatures. The upper panels in (**a,b**) show the changing environmental temperatures.

To further demonstrate the ability of the BNAs to dynamically respond to environmental temperature changes, we applied a temperature stimulus by flowing warm water (42 °C) over the surface of both the hydrogel-coated and uncoated BNAs in a microfluidic channel (see Materials and Methods). Figure 5 tracks the TE and TM mode resonant wavelengths of the two devices during temperature rising and natural cooling. For the uncoated device (Fig. 5a), the resonance shift of each resonance mode has a similar trend with the temperature variation. As the warm water arrived, the resonance shift of each peak reached a maximum value of about 3 nm for TM mode and 1.6 nm for TE mode. As the device naturally cooled down, the resonance peaks progressively blue shifted until a new temperature stimulus came. For the hydrogel-coated device (Fig. 5b), the overall resonance shift patterns are similar to those for the uncoated one, except for having a much larger amplitude at the level of 16.2 nm for TM mode and 8 nm for TE mode. Figure 5b further confirms the phase transition effect of the hydrogel around 32 °C on the resonance shift of the device. The resonant wavelength blue shifted much faster at 29–35 °C than it did in other temperatures. Therefore, the hydrogel-coated device is able to dynamically sense the environmental temperature variations and take action to shift its resonance shift.

**Figure 6:**
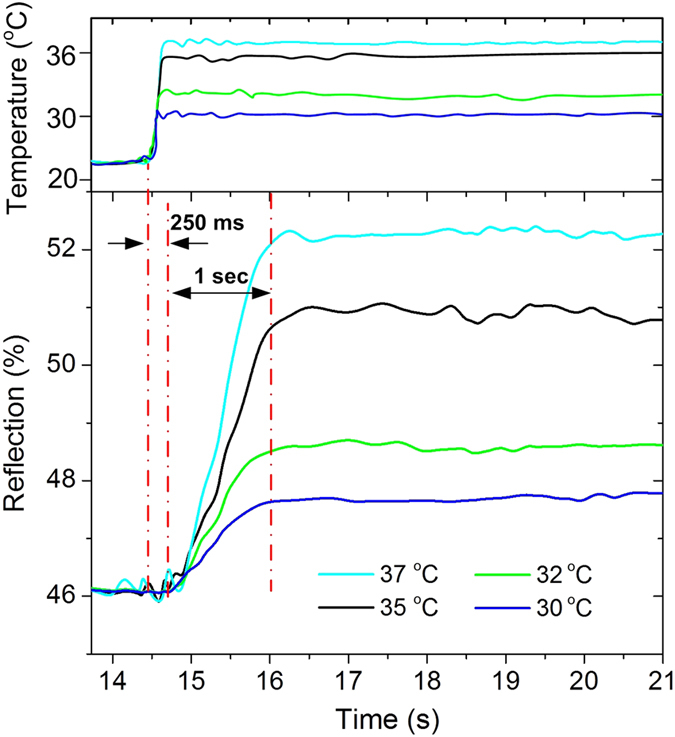
Dynamic tracking of reflection intensity of the hydrogel-coated BNAs at different temperatures. Water at different temperatures (37, 35, 32, and 30 °C) flowed over the surface of the BNAs located on the bottom of a microfluidic channel.

To quantify how fast the hydrogel-coated BNAs respond to temperature changes, we tracked changes in reflection intensity of the hydrogel-coated BNAs at the TM mode wavelength of 847 nm at 22 °C. In this study, warm water at a raised temperature (i.e., 30, 32, 35, or 37 °C) was continuously injected into the channel such that the surface temperature of the BNAs remained constant. As the warm water flowed over the device, the reflection intensities at the two fixed wavelengths reached maximum or plateau values in just about one second (Fig. 6). It should be pointed out that a response time of only 250 ms was observed for the device; this refers to the time from being exposed 42 °C warm water to a clear intensity change shown on the spectrometer. Such a short response time is attributed to the use of the submicron hydrogel coating, because the time response of the volume change approximately follows the square of the dimension as the hydrogel structure reversibly expands and contracts, depending on the temperature of the surrounding environment. As the temperature was kept constant, the hydrogel remained in a contracted state where no volumetric change of the hydrogel occurred. As a result, the resonant wavelength remained unchanged, forming the intensity plateau.

While this study utilizes the PNIPAAm hydrogel with a fixed VPTT as a model hydrogel to proof the concept of stimuli-responsive BNAs, the use of multiple thermoresponsive hydrogels with different VPTTs for multiple temperature-responsive BNAs will make it feasible to program the response of each individual BNAs device where a specific hydrogel coating is used^38^. This will provide adequate flexibility in the design of stimuli-responsive BNAs. Furthermore, a variety of hydrogels can be used to further diversify the tuning mechanisms and their applications^39^. For example, functionally complex BNAs can be realized to act as biological and chemical sensors to detect multi-environmental parameters, and subsequently generate optical outputs (resonant wavelength, and optical intensity). By working in the scale range of submicron for stimuli-responsive hydrogels where ion diffusion pathway is favorably short, and by working in the scale range of nanometers for BNAs where the localized field is sensitive to small local index changes, the stimuli-responsive BNAs will bridge local environmental input parameters with optical resonance outputs through the use of stimuli-responsive hydrogels.

## Conclusions

In this work, we have demonstrated a temperature-responsive BNAs device by coating the plasmonic dimers with a submicron-thick thermoresponsive hydrogel. Upon the temperature variations, the water content of hydrogel varies due to the transition of hydrogel from hydrophobic to hydrophilic state and will gradually alter the refractive index of hydrogel. Because of the large field enhancement of the plasmonic modes in the BNAs, the spectra shift of resonances can indicate refractive index changes. The experiment results show that for the hydrogels-coated BNAs, a 16.2 nm of resonance shift was observed, compared to a 3 nm shift for the uncoated bare BNAs. Our study suggests a possibility of making environmental-sensitive plasmonic devices through the incorporation of coupled plasmonic nanostructures and environmental-responsive materials.

## Materials and Methods

The NIPAAm hydrogel precursor solution was prepared according to the recipe described in ref. 34. The hydrogel solution contained 14.3 wt% NIPAAm, 2 wt% crosslinking agent N,N^′^-methylenebis (acrylamide) (99%) (BIS), and 2 wt% photoinitiator 2-hydroxy-4^′^-(2-hydroxyethoxy)-2-methyl propiophenone (98%) in distilled water (all purchased from Sigma-Aldrich).

The BNAs were fabricated on a 25 × 25 × 0.42 mm^3^ ITO-coated glass slide. E-beam lithography was used to form nanopatterns of BNAs in poly(methyl methacrylate) resist (Sigma-Aldrich). The area of the BNAs is 500 × 500 μm^2^ with a periodicity of 428 nm in each direction. Each bowtie has a length of 150 nm and a tip-to-tip distance of 20 nm. A 5 nm thick titanium adhesion layer and a 50 nm thick Au layer were evaporated onto the sample using an e-beam evaporator. Subsequently, a lift-off process was used to remove the metal from the regions where the e-beam resist remained. The sample was immersed into pure acetone with sonication for 20 mins. Therefore, the Au BNAs were formed.

To coat the NIPAAm hydrogel on the BNAs, a shallow air cavity was created between the ITO-coated glass slide and a polyethylene terephthalate (PET) slab. Here, a 750 nm thick photoresist was spin-coated and patterned on the glass slide to form multiple spacers. The PET slab was supported by these photoresist spacers. Therefore, the 750 nm thick air cavity was formed. Subsequently, the hydrogel precursor solution was injected into the cavity at the edge of the cavity by using a pipette. The sample was cooled down on a cooling stage with a surface temperature of 5 °C, and then, was exposed to UV light (wavelength: 365 nm, intensity: 74 mW/cm^2^) for 5 s. The low-temperature exposure enabled enhancing optical transparency of the hydrogel. Lastly, the PET slab was peeled off and nonpolymerized residual monomer was removed by rinsing the sample with ethanol and water.

Optical spectra of the sample were measured using a spectroscopic measurement setup. The incident light was coupled from a 150 watts quartz halogen lamp using a multimode fiber and focused on to the BNAs by a 60× objective lens (NA = 0.85). A polarizer was inserted between the light source and the objective to control the polarization state of the excitation light.

Optical full wave simulation was carried out using a finite element method based commercial package COMSOL Multiphysics. The geometric parameters of the BNAs were extracted from the SEM image of the fabricated device. The curvature radius at the triangle apex was set to 14 nm. The glass substrate was considered to have an infinite thickness. The 25 nm thick ITO layer between the substrate and BNAs was also included in the model of the device.

To facilitate changing environmental temperatures, a microfluidic channel (1 mm wide, 750 μm high, and 15 mm long) was built on the top of the BNAs. To form the channel, a 100 μm thick glass coverslip (Sigma-Aldrich) was placed 750 μm above the BNAs with double sided adhesive as spacers. A photopatternable polymer solution consisting of isobornyl acrylate, tetraethylene glycol dimethacrylate, and 2,2-dimethoxy-2- phenylacetophenone (all purchased from Sigma-Aldrich) with a weight ratio of 32:1.7:1^38^, was injected into the chamber formed between the coverslip and the device surface using a pipette. A film photomask (Fineline Imaging) was used to define the patterns. The UV light intensity was set to 8.4 mW/cm^2^. After 20 s exposure, the channel was developed by soaking the device in pure ethanol (Sigma-Aldrich) for 2 min, followed by baking on a hotplate at 60 °C for 1 hr. The inlet and outlet of the chambers were punched through the glass slides by using a conventional milling machine. Water with different temperatures were injected into the channel and flowed over the top surface of the device. The local temperature was monitored by a thermocouple probe (Omega HH506RA multilogger thermometer) placed in contact with the surface of the device.

## Additional Information

**How to cite this article**: Wang, Q. *et al.* Tunable Optical Nanoantennas Incorporating Bowtie Nanoantenna Arrays with Stimuli-Responsive Polymer. *Sci. Rep.* **5**, 18567; doi: 10.1038/srep18567 (2015).
